# Optimizing nitrogen application strategies can improve grain yield by increasing dry matter translocation, promoting grain filling, and improving harvest indices

**DOI:** 10.3389/fpls.2025.1565446

**Published:** 2025-04-30

**Authors:** Chuanliang Li, Yu Shi, Zhenwen Yu, Yongli Zhang, Zhen Zhang

**Affiliations:** State Key Laboratory of Wheat Improvement, Agronomy College of Shandong Agricultural University, Tai’an, China

**Keywords:** nitrogen application, wheat, photosynthetic, dry matter transport, grain yield

## Abstract

Nitrogen application enhances the grain yield of winter wheat by improving its physiological activity, dry matter production, and grain filling. However, reconciling nitrogen inputs using conservation irrigation remains challenging in water-limited wheat systems. A two-year field experiment was conducted during the 2020–2022 growing seasons with four nitrogen treatments (0 kg ha^−1^, N0; 150 kg ha^−1^, N150; 210 kg ha^−1^, N210; and 270 kg ha^−1^, N270). The responses of the senescence, dry matter accumulation and transfer, grain-filling, and grain yield of wheat to the nitrogen application rate were studied. The SPAD value, photosynthetic capacity, and antioxidant capacity of N210 flag leaves were not significantly different from those of N270 between 7–28 d after anthesis. However, these parameters were significantly higher in the N210 group than in the N0 and N150 groups. N210 and N270 significantly increased the sucrose content and sucrose phosphate synthase (SPS) activity in flag leaves relative to N0 and N150. Nitrogen application had a significant impact on dry matter transport within plants. Compared to N0, N150, and N270, dry matter transport in N210 wheat increased by 541.60–811.44 kg ha^−1^, 165.07–173.49 kg ha^−1^, and 179.02–216.74 kg ha^−1^, respectively, after anthesis. N210 significantly extended the active grain-filling period, leading to an increased grain weight. At maturity, the grain dry matter distribution in N210 was significantly higher than that in the other treatments, resulting in grain yield increases of 70.10%, 11.16%, and 6.81% compared to N0, N150, and N270, respectively. Therefore, under supplemental irrigation conditions in the North China Plain, moderate nitrogen reduction to 210 kg N ha^−1^ (N210) enhanced grain yield by delaying flag leaf senescence, improving dry matter remobilization, and optimizing grain-filling processes. The findings provide novel insights into the physiological mechanisms through which maintaining plant cellular physiological activity enhances crop productivity.

## Highlights

- Reduced nitrogen fertilizer application maintains flag leaf photosynthetic capacity and antioxidant capacity.
- Nitrogen application at 210 kg ha^−1^ increased the dry matter translocation and improved the harvest index.
- Nitrogen application at 210 kg ha^−1^ promoted grain filling, increased grain weight, and increased yield.

## 1 Introduction

The North China Plain (NCP) is the most important wheat-producing region in China, accounting for 70% of the nation’s total wheat production and 60% of its planted area (Gao et al., 2024; Liu et al., 2024). Smallholders in the NCP usually pump groundwater for three to five irrigations during the wheat season and apply 270–400 kg N ha^−1^ to wheat (Xiao et al., 2023; Abubakar et al., 2022). The unsustainable use of nitrogen fertilizers results in leaching and nitrogen loss to the environment, posing considerable risks (Wang et al., 2024). Meanwhile, the region is experiencing a severe decline in water resources, with approximately 30% of the annual precipitation, ranging from 60 to 180 mm, occurring during the winter wheat season. This amount meets only 25–40% of the water requirements for optimal wheat cultivation, further constraining agricultural productivity (Si et al., 2020).

In wheat, the dry matter accumulated in grains primarily originates from two sources, namely, post-anthesis photosynthetic assimilation and translocation of assimilates stored in vegetative organs. The post-anthesis assimilative capacity of leaves is a key determinant of final grain yield (Li et al., 2023; Fan et al., 2024). Optimized nitrogen (N) fertilization can strongly enhance plant photosynthetic capacity and dry matter accumulation (Hao et al., 2023). The photosynthetic activity duration is strongly correlated with leaf senescence (Lv et al., 2017). A moderate delay in senescence initiation can improve dry matter accumulation and its translocation to post-anthesis grains. However, excessive nitrogen application may induce the over suppression of senescence, thereby impeding the remobilization of dry matter from vegetative organs to grains (Luo et al., 2021). Compared with other source organs, flag leaves play a dominant role in determining grain yield (Noor et al., 2023). Optimized nitrogen management substantially enhances the antioxidant capacity of wheat flag leaves and decelerates their senescence process (Gaju et al., 2014). Nitrogen-mediated modifications in leaf senescence influence grain yield responses and affect grain plumpness (Luo et al., 2021). The grain-filling stage represents the second peak period of nitrogen uptake in high-yield wheat, during which it is critical to maintain adequate soil nitrogen availability (Liu et al., 2021; Ru et al., 2024). Insufficient nitrogen supply accelerates the degradation and senescence of vegetative organs during grain filling (Ma et al., 2022). Conversely, excessive nitrogen application may delay crop maturation and adversely affect the grain-filling processes (Liang et al., 2017; Fang et al., 2024). Therefore, investigating the effects of the nitrogen application rate on wheat grain-filling characteristics is of considerable theoretical and practical importance. The objective was to balance the nitrogen supply and demand, achieving high-efficiency production while reducing costs.

In agricultural production, water supply has a critical role in enhancing crop yield. However, winter wheat production in the NCP faces severe constraints owing to insufficient spring rainfall, excessive groundwater exploitation, and water scarcity. Therefore, various irrigation systems and technologies have been implemented to improve wheat yield and water use efficiency. Supplemental irrigation technology, an advanced water-saving approach refined from microsprinkler irrigation in the NCP, has substantial potential for increasing crop productivity and mitigating environmental impacts (Guo et al., 2015; Wang et al., 2015). This method offers advantages such as uniform water distribution and microclimate improvement, leading to enhancements in crop yield, water use efficiency (WUE), and nitrogen use efficiency (NUE). Our previous studies showed that under supplemental irrigation with a nitrogen application rate of 240 kg ha^−1^, grain yield, and WUE increased by 5.7–6.2% and 6.34–13.03%, respectively, compared to conventional fixed-quantity irrigation (Xu et al., 2017). Nevertheless, the optimal nitrogen application rate in this system and its effects on the physiological activity and yield of winter wheat remain unclear and require further investigation.

Nitrogen application significantly influences plant growth and development, leading to substantial variations in crop yield. In this study, we hypothesized that optimizing the fertilization strategy would improve dry matter translocation to the grain, promote grain filling, and ultimately increase grain yield. To evaluate this hypothesis, a two-year field experiment was conducted. The objectives of this study were (I) to determine the appropriate amount of nitrogen to be applied to achieve high yields under the conditions of this experiment, and (II) to identify the main drivers of yield variability in wheat by analyzing the relationship between yield and post-anthesis leaf physiological traits and grain filling characteristics. Conducted in the NCP, in this study, we aimed to establish a theoretical foundation for strategic nitrogen management practices in regional wheat production, while also providing a reference for wheat cultivation in other ecological zones.

## 2 Materials and methods

### 2.1 Research location

**Figure 1:**
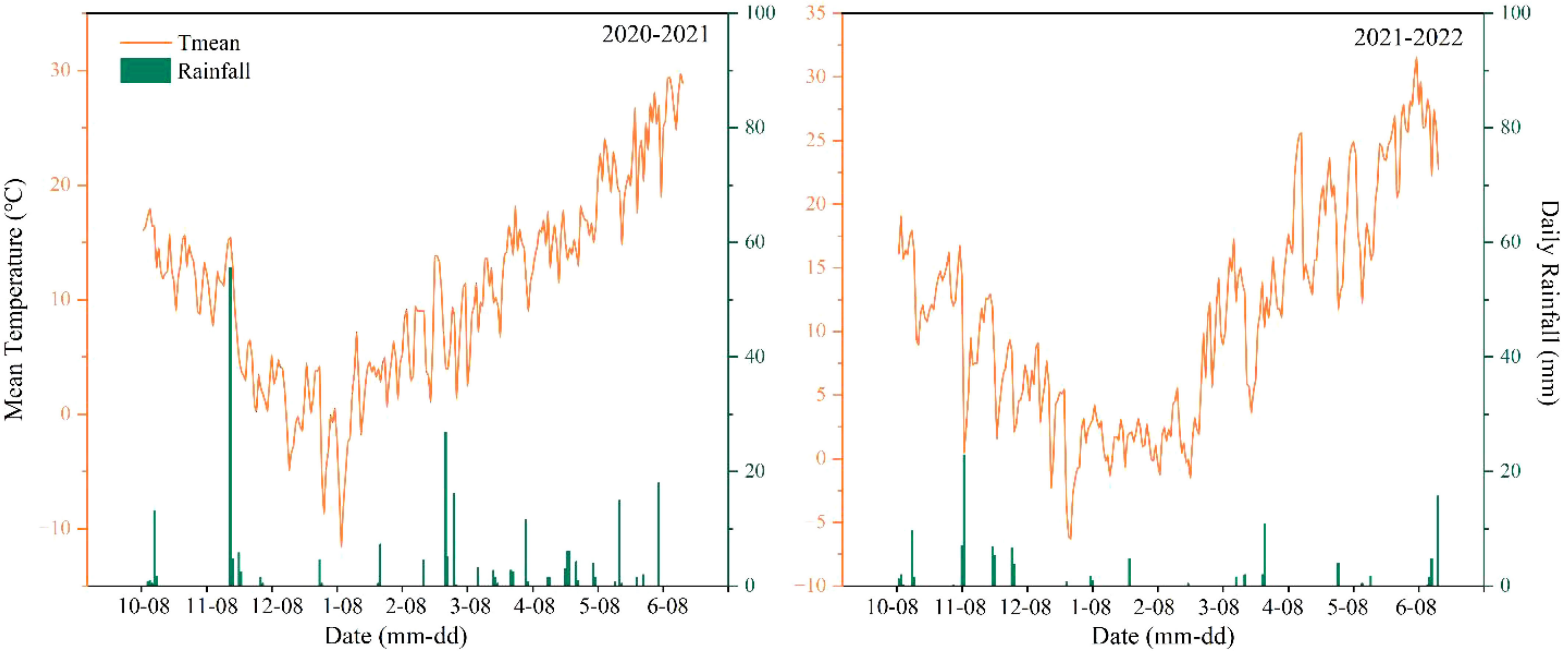
Daily rainfall (bars) and average temperature (line) during the wheat growing seasons of 2020–2021 and 2021–2022.

From 2020 to 2022, field experiments were conducted in the Yanzhou District of Jining City, Shandong Province (35°40′N, 116°41′E) over two growing seasons. The study area was located within a warm-temperate continental semi-humid monsoon climate zone with an average annual temperature of 13.5°C, an average annual sunshine duration of 2,567 h, and an average annual precipitation of 703 mm. The average daily rainfall and temperature during the two growing seasons are shown in **Figure 1**. The previous crop in the experimental field was maize. N fertilizer (N), phosphate fertilizer (P_2_O_5_), and potassic fertilizer (K_2_O) were applied before the maize was sown at 240 kg ha^−1^,90 kg ha^−1^, and 90 kg ha^−1^, respectively. All the straw was returned to the field after the maize harvest, and the average amount of straw returned to the field was 10,000 kg ha^−1^. **Table 1** presents the physicochemical properties of the soil in the top 20 cm layer before winter wheat sowing.

**Table 1:** Soil physical and chemical properties in the 0–20 cm soil layer before sowing of winter wheat.

Organic matter (g·kg^-1^)	Total nitrogen (g kg^-1^)	Available phosphorus (mg kg^-1^)	Available potassium (mg kg^-1^)	Alkali-hydrolyzable nitrogen (mg kg^-1^)	Field water capacity (%)	Soil bulk density (g·cm^-3^)
14.22	1.02	32.25	116.9	121.79	29.9	1.49

### 2.2 Experimental design and field practices

The high-yielding winter wheat (Triticum aestivum L.) variety, ‘Yannong1212’, was planted over two consecutive growing seasons. ‘Yannong1212’ is a conventional variety of semi-wintering, semi-prostrate seedlings, and forms semi-compact plants. Seeds were sown using a planter with wide spacing (2BJK-8; Yuncheng Gongli Co., Ltd., Heze, China), with a sowing band of 8 cm and row spacing of 25 cm. The sowing dates were October 10, 2020, and October 22, 2021, with harvests taking place on June 8, 2021, and June 7, 2022, respectively.

The experiment was designed with four nitrogen (N) application rates: 0 kg N ha^−1^ (N0), 150 kg N ha^−1^ (N180), 210 kg N ha^−1^ (N210), and 270 kg N ha^−1^ (N270). Basal N fertilizer was applied before sowing, and follow-up N fertilizer was applied at a jointing ratio of 5:5. Each treatment was fertilized with 135 kg ha^−1^ P_2_O_5_ and 150 kg ha^−1^ K_2_O as base fertilizer. The plot area was 30 m^2^ (2 × 15 m). Each treatment was replicated thrice. Water management of the experimental field was conducted through supplemental irrigation, where the relative water content of the 0–40 cm soil layer was supplemented to 70% of the field water-holding capacity at the stage of wheat jointing and anthesis. The amount of irrigation was calculated according to the formula outlined by Man et al. (2014) (Equation 1):


(1) M=10×r×H× (βi−βj)

The planned wetted soil depth (H), the average soil bulk weight at the planned wetted soil depth (r), the target soil mass moisture content (βi), and the pre-irrigation soil mass moisture content (βj) were the variables in question. **Supplementary Table S1** provides a detailed description of the fertilizer and irrigation regimes employed in this study. In addition to these inputs, other field practices, such as pesticide or herbicide application, were conducted manually following local farming practices.

### 2.3 Grain yield and dry matter accumulation

The total number of spikes was recorded during the prewinter, jointing, anthesis, and mature stages. At the mature stage, 50 spikes were randomly selected and the number of grains per spike was determined. Plants from three representative 3 m^2^ samples were selected in each plot, threshed, and weighed after natural drying. The moisture content was converted uniformly to 13% to determine the grain yield. The 1000-grain weight was determined in triplicate (Liu et al., 2023).

Twenty uniformly growing wheat plants were randomly selected from each experimental plot at anthesis, and maturity and their dry matter weights were measured. The plants were decomposed into stems, leaves, and spikes at the anthesis stage, and into stems, leaves, grains, and the remaining parts at maturity. The plants were weighed after being dried at 75°C to a consistent weight. Post-anthesis dry matter accumulation was estimated by subtracting the dry matter accumulation at anthesis and maturity. The ratio of grain yield to the total aboveground dry matter accumulated at maturity was used to construct the harvest index (Yan et al., 2022).

### 2.4 Flag leaf SPAD and photosynthetic properties

Both data were recorded only for the 2021–2022 growing season. To assess leaf greenness, the chlorophyll content (SPAD) was measured using a portable CCM-200 Plus Chlorophyll Content Meter (Opti-Sciences Inc., NH, USA) from 40 representative flag leaves at 0, 7, 14, 21, and 28 d after anthesis. Twenty flag leaves in each experimental plot were sampled at 7-d intervals from anthesis to maturity to obtain the net photosynthetic rate (Pn), transpiration (Tr), and stomatal conductance (Gs) using a LI-6400 Portable Photosynthesis System (Li-Cor, Lincoln, USA). The measurements were performed from 9:00 to 11:00 AM under natural light (He et al., 2020).

### 2.5 Flag leaf senescence

Flag leaves of uniformly growing wheat plants were labeled for each treatment at anthesis. Fresh flag leaves were immediately immersed in liquid N and then stored at −80°C until biochemical assays had been performed. For enzyme extraction, fresh flag leaf samples were cut into 0.5 g pieces, ground in a mortar and pestle with liquid N, and extracted in 5 mL of potassium phosphate buffer solution (pH = 7.8) containing 0.2 mol L^−1^ KH_2_PO_4_ and 0.2 mol L^−1^ K_2_HPO_4_. The homogenate was centrifuged at 10,000 × g for 20 min at 4°C, and then the supernatant was obtained for enzyme analysis (Kong et al., 2017; Liu et al., 2019; Lv et al., 2021).

Superoxide dismutase (SOD) activity was assayed by measuring the inhibition of nitro blue tetrazolium (NBT). A 3 mL reaction mixture contained 50 μM NBT, 13 mM methionine, 75 μM NBT chloride, 0.1 mM EDTA, 50 mM phosphate buffer (pH 7.8), 50 mM sodium carbonate, and 0.1 mL crude extract. Test tubes containing the reaction mixture were placed under a light bank (15 fluorescent lamps) delivering 78 μmol m^−2^ s^−1^ for 15 min. Absorbance was measured at 560 nm using a spectrophotometer (Hitachi U-1100, Tokyo, Japan). One unit of SOD activity was defined as the amount of enzyme required to inhibit NBT photoreduction by 50%. MDA content was determined spectrophotometrically through its reaction with 0.5% thiobarbituric acid at wavelengths of 532 and 600 nm. The formula used for the calculation was A = (D532–D600)/(E × m), where A represents the MDA concentration, D is the optical density, m is the plant wet biomass, and E is a constant coefficient. The soluble protein content was determined using Komas Brilliant Blue (G-250) staining.

### 2.6 Flag leaf sucrose content and SPS activity

The sampling method is the same as that described in Section 2.5. 1 g of leaves was added with Hepes-NaOH buffer (pH = 7.5) 1 mL, ground in an ice bath, and centrifuged at 10,000×g for 10 min, and the supernatant was the enzyme solution. Anthrone colorimetry was used to determine the sucrose content in flag leaves. Flag leaf sucrose phosphate synthase (SPS) activity was determined using the resorcinol method (Guo et al., 2015).

### 2.7 Grain-filling traits

From anthesis to maturity, 40 spikes were taken from each plot at 7-d intervals. The grain-filling rate was determined from the accumulated dry weight. At each sampling date, kernels were separated from the glumes and stalks to determine grain dry weight. The total number of kernels was determined, and dry weights were recorded. Logistic equations for fitting the grain-filling process and filling parameters were proposed by He et al. (2020) (Equation 2).


(2) $$\text{y}=\text{a}/\left(1+{\text{be}}^{-\text{cx}}\right)$$

where y (mg) is the kernel weight, a (mg) is the upper asymptote of the kernel weight, b and c are coefficients determined by the curvature, and x (d) is the grain-filling time. The following equations were used to calculate the time required to reach the maximum grain-filling rate (Tmax, Equation 3): the duration of grain filling (T, Equation 4), defined as the period when y was achieved at 99% of a; the grain weight at the maximum grain-filling rate (Wmax, Equation 5), maximum grain-filling rate (Vmax, Equation 6), and active grain-filling period (D, Equation 7), defined as the period when y was between 5% and 95% of a:


(3) Tmax=lnb/c

(4) T=(lnb+4.59512)/c

(5) Wmax=a/2

(6) Vmax=c×Wmax×(1−Wmax/a)

(7) D=6/c

### 2.8 Statistical analyses

Normality and homogeneity of variance were tested using the Shapiro–Wilk test (p>0.05) and Levene’s test (p > 0.05), respectively, before statistical analyses (SPSS 19.0; SPSS Inc., Chicago, USA). Any dataset that did not conform to a normal distribution was squared or log10 transformed to conform to the assumption of normality before further statistical analyses. One-way analysis of variance (ANOVA) and Fisher’s Least Significant Difference (LSD) test (p<0. 05) were used to investigate the effects of different fertilizer combinations on the grain yield, photosynthetic capacity, antioxidant capacity, sucrose metabolism, seed filling, and dry matter accumulation. Histograms and dotted line plots were generated using Origin 2021 (Origin Lab Inc., USA) to illustrate the differences between treatments.

## 3 Results

### 3.1 Flag leaf photosynthetic capacity

**Figure 2:**
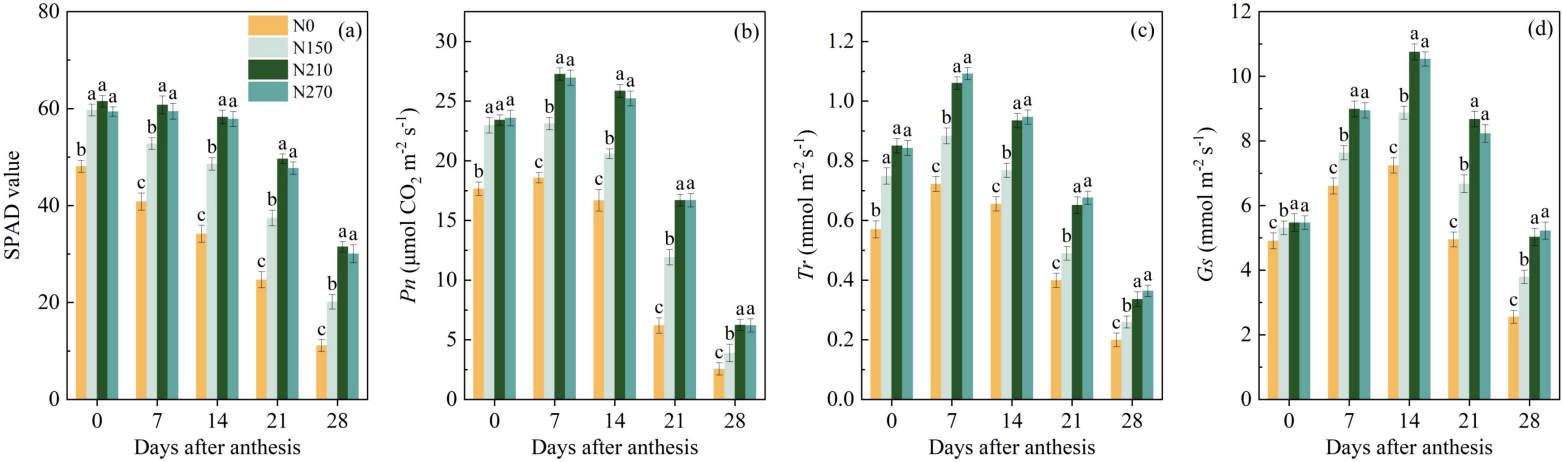
Effect of nitrogen application on SPAD values **(a)**, net photosynthetic rate (*Pn*, **b**), transpiration rate (*Tr*, **c**) and stomatal conductance (*Gs*, **d**) of flag leaves after anthesis. **(a–d)** represent results from the 2021–2022 growing season. Different lowercase letters denote significant differences (ANOVA, Fisher’s least significant difference test, P<0.05). N0, N150, N210 and N270, the nitrogen application rates are 0, 150, 210, and 270 kg·ha^–1^, respectively.

The nitrogen application rate significantly influenced the SPAD values and photosynthetic capacity of wheat flag leaves during the post-anthesis period (**Figure 2**). At anthesis, no significant differences were observed in the flag leaf SPAD values or net photosynthetic rate (Pn) among the N150, N210, and N270 treatments. However, these values were significantly higher than those in the N0 treatment. From 7 to 28 d after anthesis (DAA), the N210 treatment showed 31.36% and 20.62% higher average SPAD values and Pn compared to N150, respectively. However, further increasing the nitrogen application to 270 kg ha^−1^ (N270) did not result in significant changes in SPAD values. Similarly, the transpiration rate (Tr) and stomatal conductance (Gs) of flag leaves followed trends comparable to those of SPAD and Pn leaves. During 7–28 DAA, no significant differences in Tr or Gs were detected between the N210 and N270 treatments. However, Tr and Gs remained significantly higher than those in the N150 and N0 treatments.

### 3.2 Antioxidant capacity of flag leaf

**Figure 3:**
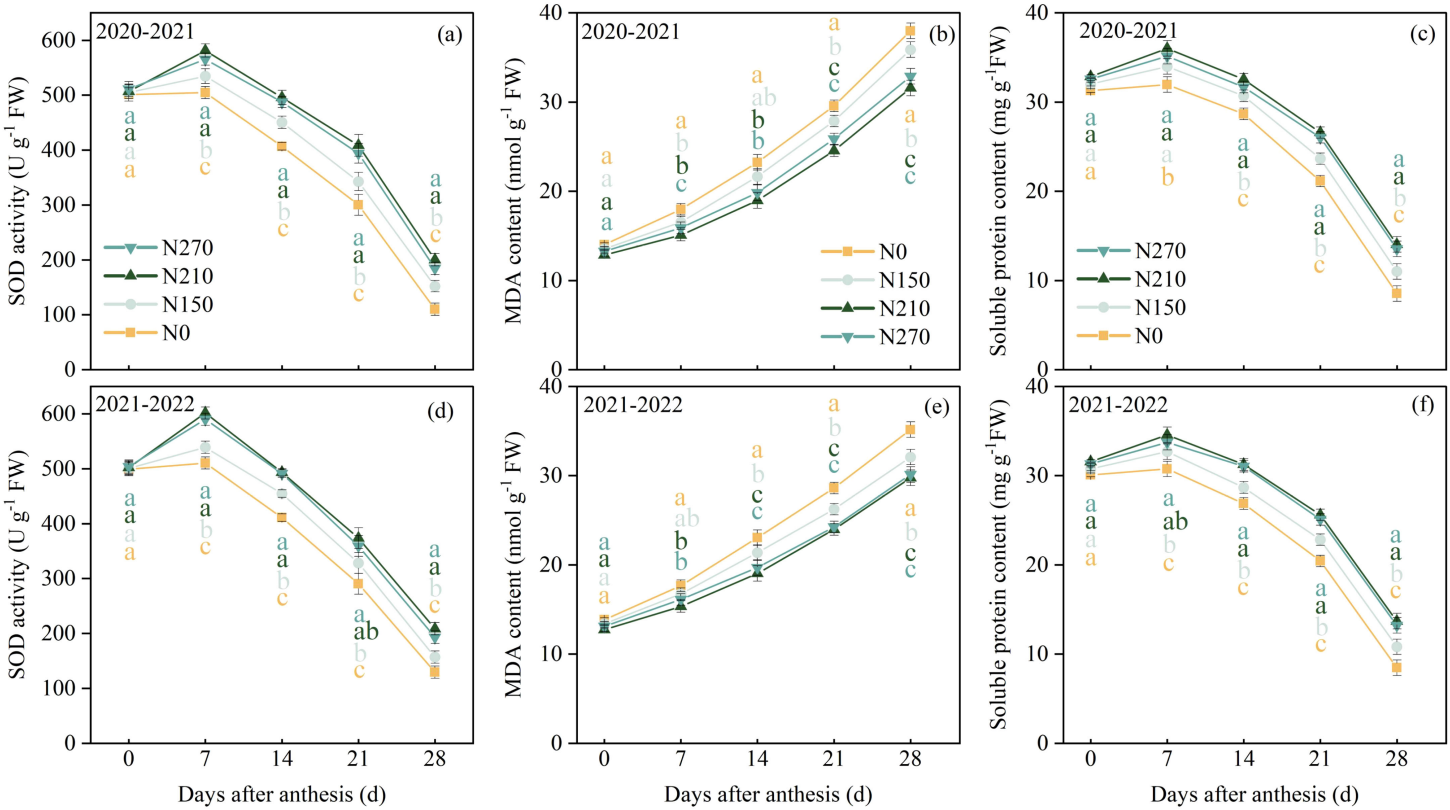
Effects of nitrogen application on SOD activity **(a, d)**, malondialdehyde (MDA) content **(b, e)**, and soluble protein content **(c, f)** in wheat flag leaves after anthesis. Different lowercase letters denote significant differences (ANOVA, Fisher’s least significant difference test, P<0.05). For N0, N150, N210, and N270, the nitrogen application rates are 0, 150, 210, and 270 kg·ha^−1^, respectively.

As shown in **Figure 3**, no significant differences were observed in SOD activity or soluble protein content among the treatments at anthesis. From 7 to 28 DAA, the N210 and N270 treatments maintained significantly higher SOD activity and soluble protein content than the N0 and N150 treatments. Malondialdehyde (MDA) content in wheat flag leaves increases progressively during the post-anthesis phase. No significant differences were detected between the N210 and N270 treatments from 14 to 28 DAA. However, both showed lower MDA content compared to the N150 and N0 treatments during this period.

### 3.3 Sucrose content and SPS activity

**Figure 4:**
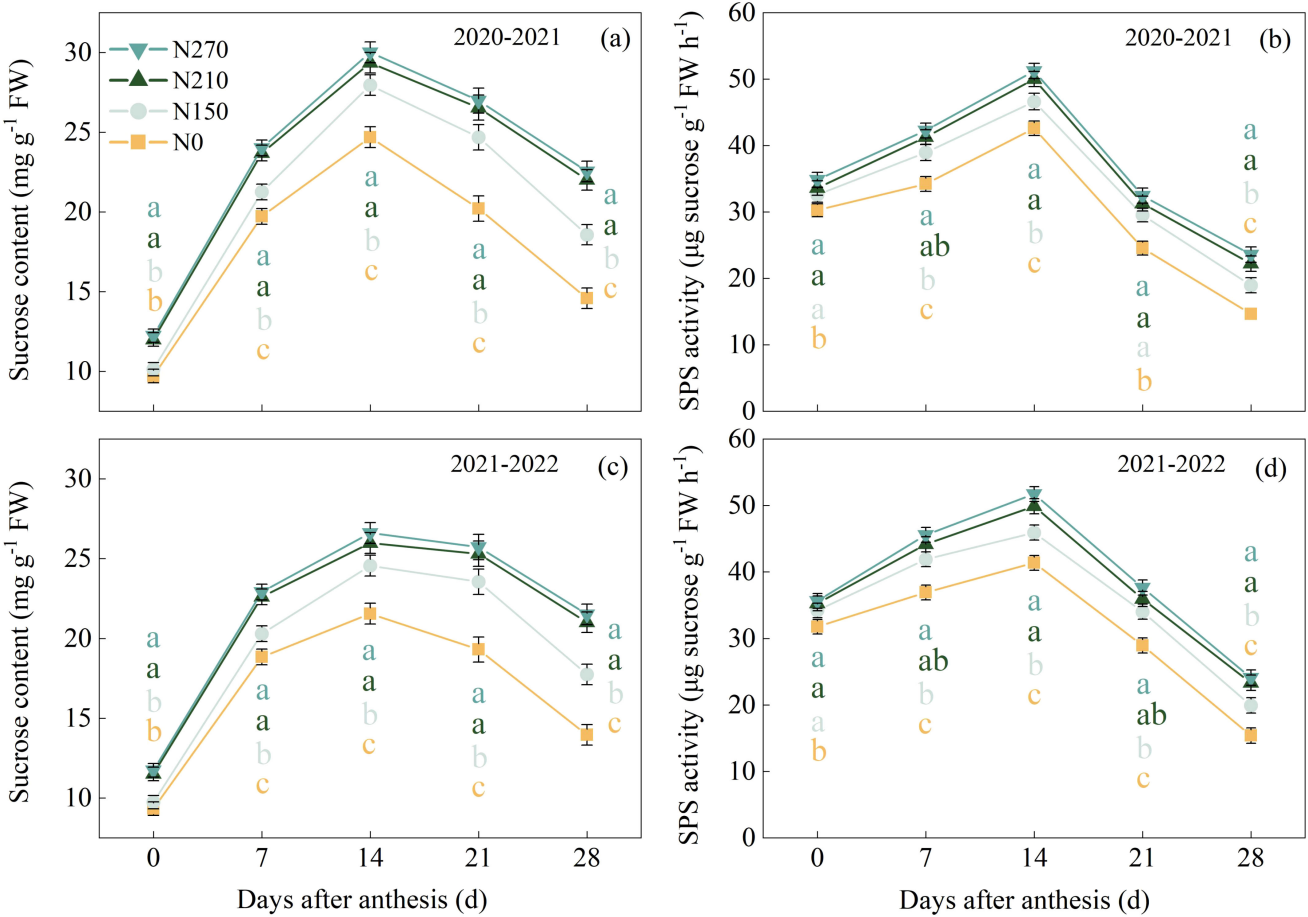
Effects of nitrogen application on sucrose content **(a, c)** and phospho-sucrose synthase (SPS) activity **(b, d)** in wheat flag leaves after anthesis. (**a–d**) represent the results for the growing seasons 2020–2021 and 2021–2022, respectively. Different lowercase letters denote significant differences (ANOVA, Fisher’s least significant difference test, P<0.05). For N0, N150, N210, and N270, the nitrogen application rates are 0, 150, 210, and 270 kg·ha^−1^, respectively.

The nitrogen application rate significantly influenced sucrose content and SPS activity in wheat flag leaves (**Figure 4**). During the 2020–2021 growing seasons, the N210 treatment reached peak sucrose levels (29.35 mg g^−1^ FW) 21 DAA, representing 143.56% and 23.89% increases, respectively, compared to the N0 (12.05 mg g^−1^ FW) and N150 (23.69 mg g^−1^ FW) treatments. Excessive nitrogen application (N270) did not yield a further significant enhancement in sucrose content. SPS activity showed strong temporal coordination with sucrose accumulation dynamics (**Figures 4b, d**). In both growing seasons, the N210 and N270 treatments maintained elevated SPS activity from 7 to 28 DAA, with no significant differences observed between the two nitrogen levels during this period.

### 3.4 Grain-filling characteristics

**Figure 5:**
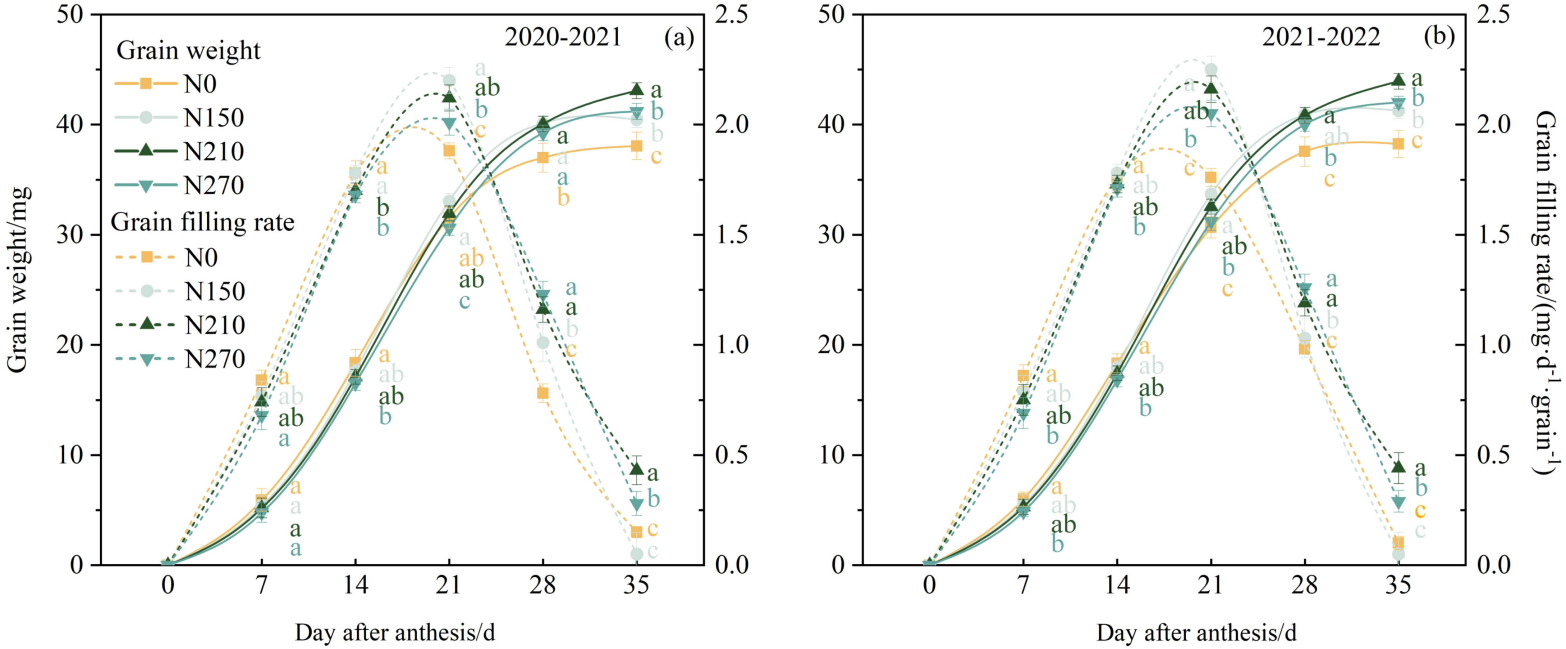
Effects of nitrogen application on grain weight and grain filling rate of wheat after anthesis. The solid line represents grain weight, and the dashed line represents grain filling rate. **(a, b)** represent results for the 2020–2021 and 2021–2022 growing seasons, respectively. Different lowercase letters denote significant differences (ANOVA, Fisher’s least significant difference test, P<0.05). For N0, N150, N210, and N270, the nitrogen application rates are 0, 150, 210, and 270 kg·ha^−1^, respectively.

Throughout the two growing seasons, the wheat grain-filling process under different nitrogen fertilizer treatments exhibited “S”-shaped changes (**Figure 5**), with the filling rate following a single-peak curve. From 0 to 14 DAA, no significant differences in grain weight were observed between the nitrogen application treatments. From 28 to 35 DAA, the nitrogen-treated plots showed significantly higher grain weights than the N0 treatment plots. A logistic equation was used to model the effects of nitrogen application on the grain-filling rate of wheat (**Table 2**). The N210 treatment resulted in a longer maximum grain-filling rate and active grain-filling period. This suggests that the 210 kg ha^−1^ nitrogen application delayed the maximum filling rate and prolonged the peak filling period, which favored an increase in grain weight.

**Table 2:** The effect of nitrogen application rate on grain filling parameters.

Treatment		Growth curve equation	T_max_/d	T/d	W_max_/ (mg·grain^−1^)	V_max_/ (mg·grain^−1^·d^−1^)	D/d
2020–2021	N0	Y = 39.24/(1 + 29.75e^−0.23x^)	14.70c	34.61c	19.62c	2.26c	26.00b
	N150	Y = 42.00/(1 + 41.15e^−0.25x^)	15.16b	33.90b	21.00b	2.57a	24.47c
	N210	Y = 44.29/(1 + 33.80e^−0.22x^)	16.18a	37.30a	22.15a	2.41a	27.58a
	N270	Y = 42.66/(1 + 35.86e^−0.22x^)	16.17a	36.94a	21.33b	2.36b	27.11a
2021–2022	N0	Y = 38.27/(1 + 31.60e^−0.24x^)	14.38c	33.52b	19.13b	2.30b	24.98b
	N150	Y = 41.18/(1 + 40.99e^−0.24x^)	15.16b	33.91b	20.59b	2.52a	24.49b
	N210	Y = 43.42/(1 + 33.68e^−0.22x^)	16.17a	37.31a	21.71a	2.36a	27.59a
	N270	Y = 41.82/(1 + 35.73e^−0.22x^)	16.16a	36.94a	20.91b	2.31b	27.12a

Tmax, time to reach maximum grain filling rate; T, duration of grain filling; Wmax, weight of maximum grain filling rate; Vmax, maximum grain filling rate; D, active grain filling period. Different lowercase letters denote significant differences (ANOVA, Fisher’s least significant difference test, P<0.05). N0, N150, N210, and N270, the nitrogen application rates are 0, 150, 210, and 270 kg·ha^−1^, respectively.

### 3.5 Dry matter accumulation and transportation

**Figure 6:**
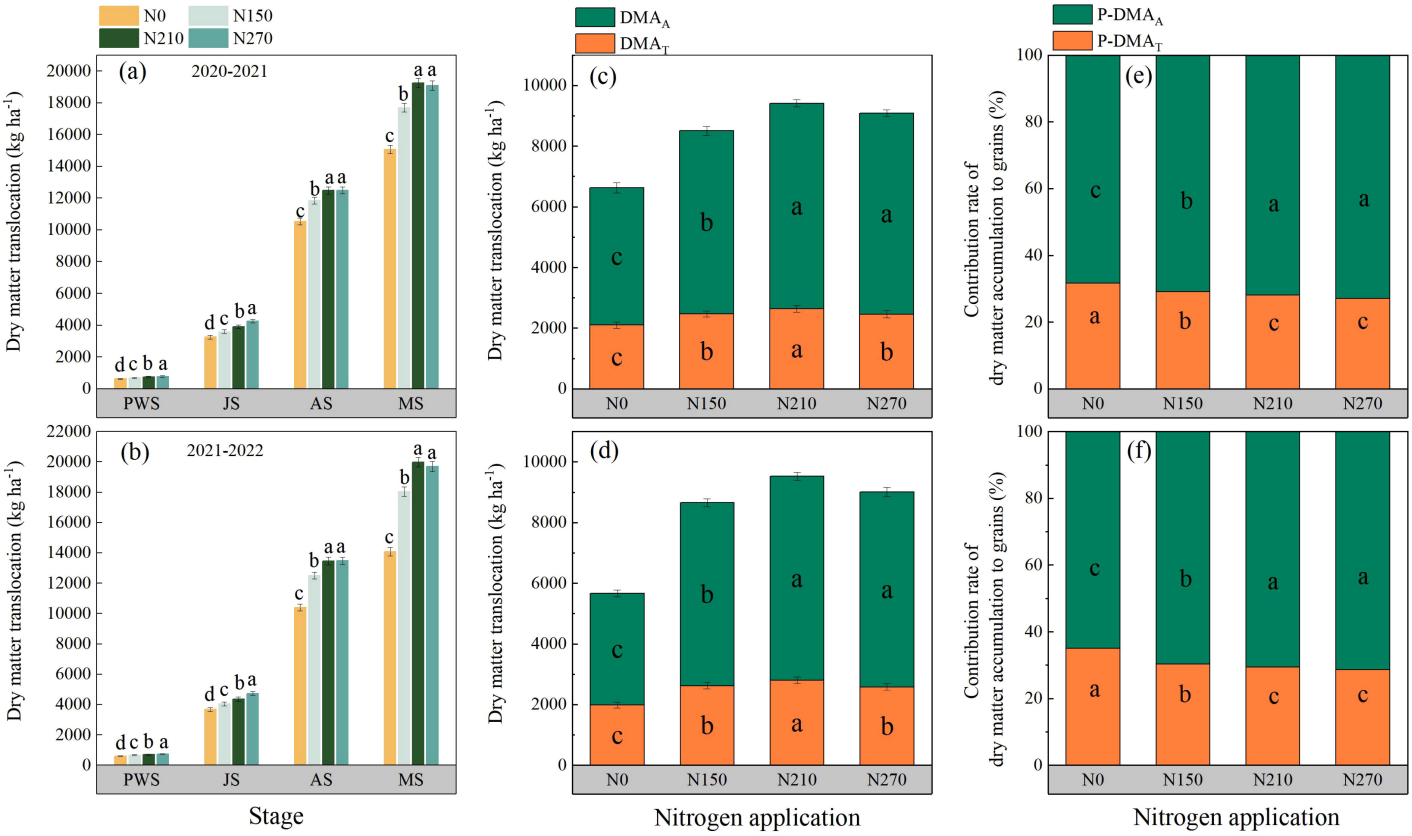
Effects of nitrogen application on dry matter accumulation **(a, b)**, dry matter translocation **(c, d)**, and contribution of dry matter translocation **(e, f)** to grain yield of wheat plants in various reproductive periods. PWS, JS, AS, and MS represent pre-winter, jointing, anthesis, and maturation, respectively. DMA_T_ and DMA_A_ represent dry matter translocation and post-anthesis dry matter accumulation, respectively. P-DMA_T_ and P-DMA_A_ represent the contribution of DMA_T_ and DMA_A_ to grain yield, respectively. **(a, c)** and **(e)**; **(b, d)** and **(f)** represent the results for the growing seasons 2020–2021 and 2021–2022. Different lowercase letters denote significant differences (ANOVA, Fisher’s least significant difference test, P<0.05). N0, N150, N210, and N270, the nitrogen application rates are 0, 150, 210, and 270 kg·ha−1, respectively.

Nitrogen application rates had pronounced effects on dry matter accumulation (DMA) across wheat growth stages (**Figure 6**). In both growing seasons, the prewinter DMA of wheat plants increased with elevated nitrogen input. A similar trend is observed during the jointing stage. The highest DMA values at anthesis (12,478.95–13,462.77 kg ha^−1^) and maturity (19,254.87–19,992.68 kg ha^−1^) were recorded under the N210 treatment. A further increase in nitrogen application to N270 did not induce significant changes in plant DMA. Across both seasons, the N210 treatment had the highest post-anthesis DMA, reaching 6,775.92 kg ha^−1^ in 2020–2021 and 6,829.91 kg ha^−1^ in 2021–2022. Compared with the N0 and N150 treatments, both N210 and N270 significantly enhanced the contribution of post-anthesis dry matter assimilation to grain yield during both growing seasons.

### 3.6 Dry matter partitioning at maturity

**Figure 7:**
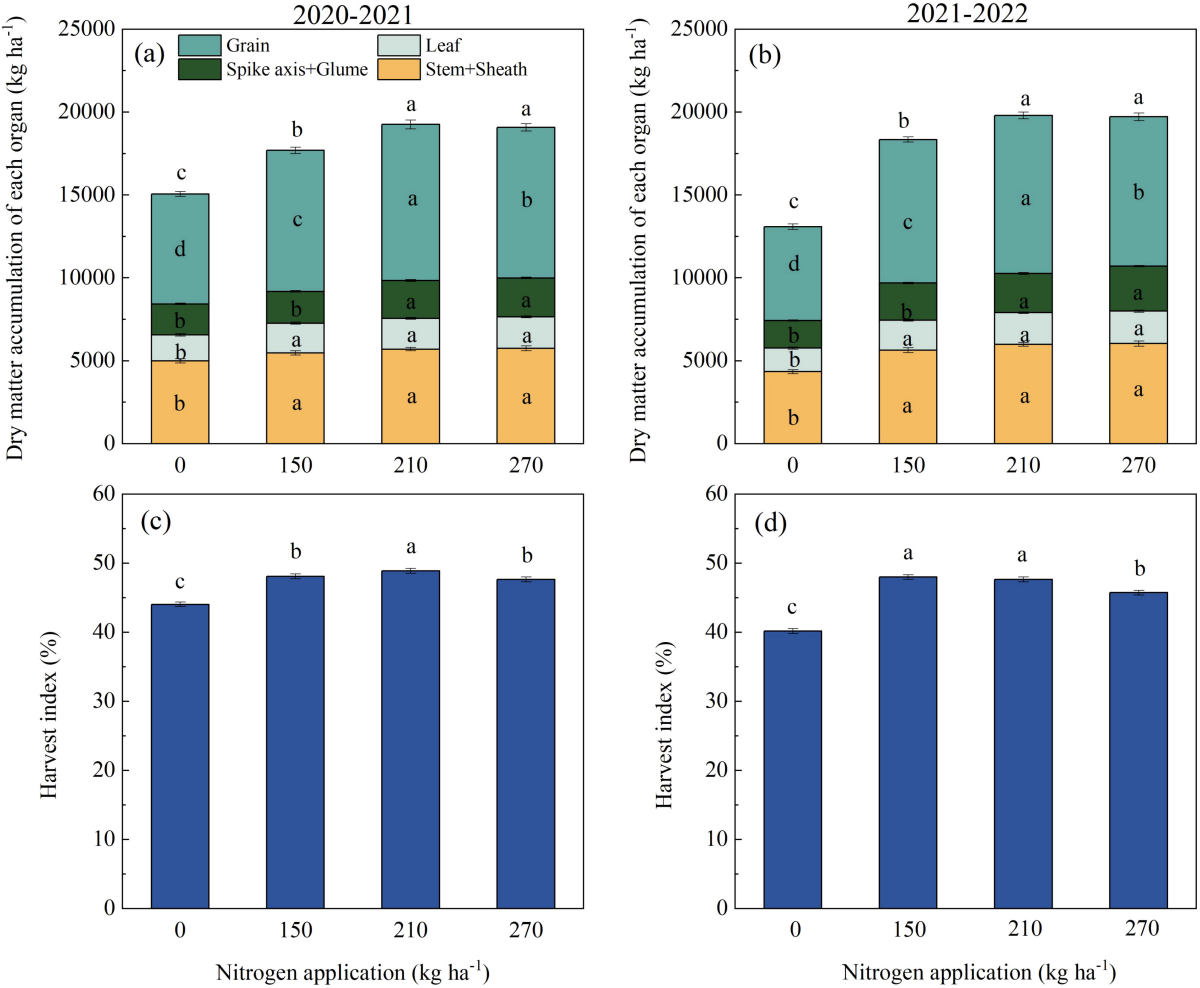
The effects of nitrogen application rate on dry matter distribution **(a, b)** and harvest index **(c, d)** of wheat during maturity. (**a, c**; **b, d**) represent the results for the growing seasons 2020–2021 and 2021–2022. Different lowercase letters denote significant differences (ANOVA, Fisher’s least significant difference test, P<0.05). For N0, N150, N210, and N270, the nitrogen application rates are 0, 150, 210, and 270 kg·ha^−1^, respectively.

Nitrogen application significantly influenced the dry matter distribution in wheat plants at maturity (**Figures 7a, b**). The DMA of grain in the N210 treatment increased by 2784.63–3867.04 kg ha^−1^ compared to the N0 treatment, by 866.31–909.98 kg ha^−1^ compared to the N150 treatment, and by 324.01–513.33 kg ha^−1^ compared to the N270 treatment. As depicted in **Figures 7c, d**, the highest harvest index was observed in both growing seasons for the N210 treatment, with values of 48.89% (2020–2021) and 47.65% (2021–2022).

### 3.7 Grain yield and yield components

Nitrogen fertilizer, year, and their interaction significantly influenced grain yield and its components (**Table 3**). In both growing seasons, the number of spikes in winter wheat significantly increased with higher nitrogen levels (N270 > N210 > N150 > N0). The highest number of grains per spike and 1,000-grain weight were observed in the N210 treatment. In the 2020–2021 growing season, N210 increased the yield by 43.57%, 10.86%, and 4.74% compared with N0, N150, and N270, respectively. Similar trends were noted in the 2021–2022 growing season, with N210 again producing the highest yield. Moderate nitrogen application enhances grain yield, whereas excessive nitrogen negatively affects grain weight per spike and 1,000-grain weight, limiting yield improvement.

**Table 3:** Effects of micro-sprinkling irrigation with different nitrogen rates and irrigation amounts on spike number, grain number per spike, 1 000-grain weight, and grain yield of winter wheat.

Treatment		Spike number (×10^4^ spike ha^−1^)	Grain number per spike	1 000-grain weight (g)	Grain yield (kg·ha^−1^)
2020–2021	N0	495.15 ± 4.98d	35.99 ± 0.83c	43.21 ± 0.18c	6629.92 ± 96.65d
	N150	575.36 ± 8.06c	38.95 ± 0.79b	44.58 ± 0.34b	8504.57 ± 95.54c
	N210	601.59 ± 9.28b	39.58 ± 0.54a	45.79 ± 0.37a	9414.55 ± 121.25a
	N270	626.08 ± 10.52a	38.58 ± 0.58b	44.01 ± 0.21b	9090.54 ± 114.52b
2021–2022	N0	505.35 ± 10.17d	31.26 ± 0.29c	41.68 ± 0.38c	5658.94 ± 98.77d
	N150	638.25 ± 10.66c	37.73 ± 1.04a	42.99 ± 0.44b	8659.64 ± 112.24c
	N210	675.35 ± 9.18b	37.65 ± 0.47a	44.01 ± 0.30a	9625.98 ± 110.56a
	N270	692.55 ± 8.04a	36.84 ± 0.55b	42.56 ± 0.37b	9012.65 ± 147.85b
Y		**	**	**	ns
N		**	**	**	**
Y × N		*	ns	*	ns

N0, unfertilized control; N150, 150 kg ha^−1^; N210, 210 kg ha^−1^; N270, 270 kg ha^−1^. Means followed by different lowercase letters within the same row within each year indicate significant differences between the different fertilization treatments (P<0.05). Two-way ANOVA for the effect of nitrogen application rate (N) and year (Y) on spike number, grain number per spike, 1 000-grain weight, and grain yield. Different lowercase letters denote significant differences (ANOVA, Fisher’s least significant difference test, P<0.05). *Significance at the 0.05 probability level. **Significance at the 0.01 probability level. NS, not significant.

### 3.8 Correlation analysis

**Figure 8:**
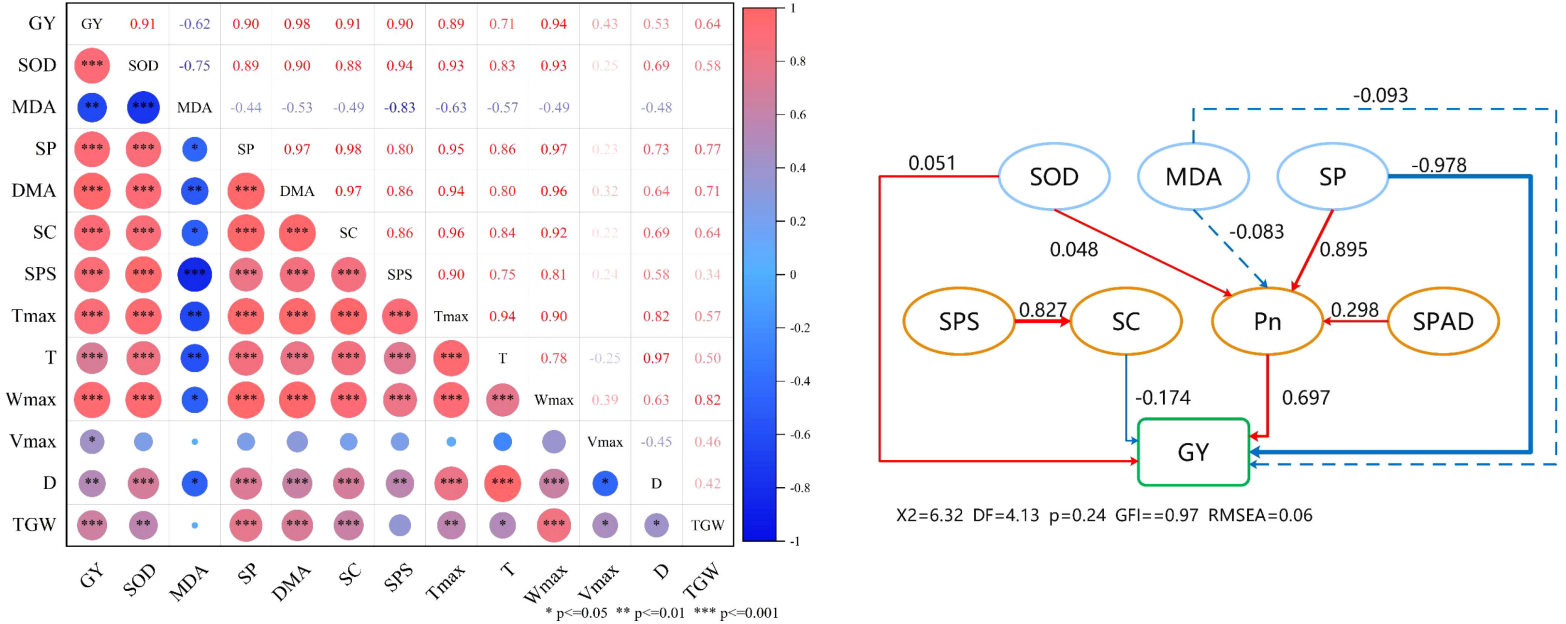
Correlation analysis **(A)** and structural equation model **(B)**. GY, grain yield; SOD, superoxide dismutase; MDA, malondialdehyde; SP, soluble protein; DMA, dry matter after anthesis; SC, sucrose content; SPS, sucrose phosphate synthase, Tmax, the days achieving the maximal grain-filling rate; T, duration of grain filling; Wmax, grain weight achieving the maximal grain-filling rate; Vmax, maximum grain–filling rate; D, active grain-filling date; TGW, 1000-grain weight. The red and blue arrows represent positive and negative relationships, and non-significant paths are represented by dashed arrows, respectively. The thickness of the arrow is proportional to the intensity of the causal effect. The number at the arrow is a standardized path coefficient or covariate coefficient.

Correlation analysis showed a highly significant positive correlation between flag leaf SOD content, soluble protein content, sucrose content, dry matter after anthesis, sucrose phosphate synthase activity, and grain yield of wheat after anthesis (**Figure 8**). There was a significant negative correlation between flag leaf MDA content and grain yield. GY positively correlated with Tmax, t, Wmax, Vmax, D, and thousand grain weight (TGW). The TGW was positively correlated with Tmax, t, Wmax, Vmax, and D.

## 4 Discussion

### 4.1 Effects of nitrogen application on photosynthetic characteristics, sucrose content, and SPS activity of wheat flag leaves

The SPAD values of wheat flag leaves exhibited a gradual decline during the post-anthesis stage, while the net photosynthetic rate (Pn), transpiration rate (Tr), and stomatal conductance (Gs) displayed a unimodal pattern characterized by an initial increase followed by a subsequent decrease (**Figure 2**). The temporal decline in post-anthesis flag leaf SPAD values reflects a coordinated senescence process involving chloroplast dismantling and chlorophyll catabolism (Wu et al., 2019). The unimodal pattern of Pn, Tr, and Gs aligns with the dual-phase regulation of photosynthesis: an initial enhancement driven by sink strength during early grain filling, followed by progressive limitations due to reduced mesophyll conductance and photosystem II (PSII) photoinhibition under declining nitrogen availability. Notably, nitrogen treatments N210 and N270 significantly increased flag leaf SPAD values and enhanced overall photosynthetic capacity (**Figure 2**). This improvement likely originates from nitrogen-mediated preservation of chloroplast ultrastructure and upregulation of chlorophyll biosynthesis enzymes, thereby prolonging the functional duration of photosynthetic apparatus during grain filling (Zhang et al., 2017). Sustained photosynthetic activity facilitated continuous carbon assimilate supply to developing grains, a critical requirement for starch deposition during mid-grain filling. However, photosynthetic capacity showed no significant improvement when nitrogen application exceeded 210 kg ha^−1^ (**Figure 2**), indicating a threshold effect in nitrogen-mediated photosynthetic enhancement. This plateau may reflect saturation of nitrogen incorporation into photosynthetic proteins or limitations in chloroplast electron transport chain components (Zhang et al., 2013).

Under N210 and N270 treatments (**Figure 4**), the observed maintenance of elevated sucrose content and sucrose phosphate synthase (SPS) activity in flag leaves provides biochemical evidence for enhanced assimilate production and partitioning. The strong positive correlations between SPAD values, Pn, sucrose content, and SPS activity (**Figure 8**) align with the source-strength model proposed by Liu et al. (2021), where sufficient nitrogen coordinates light capture, carbon fixation, and sucrose synthesis-transport processes. Remarkably, the N210 treatment optimized the temporal synchronization between sucrose synthesis peaks and grain filling demands. Elevated SPS activity during mid-grain filling (**Figure 5**) enhances sucrose flux to developing endosperm, where it serves as both carbon skeletons and energy sources for starch synthase reactions. This metabolic coordination ensures adequate substrate supply for glycolysis and subsequent starch biosynthesis, particularly during the critical window of grain dry matter accumulation (Lv et al., 2021). Our findings demonstrate that nitrogen management at 210 kg ha^−1^ achieves an optimal balance between photosynthetic capacity maintenance and assimilate partitioning efficiency in wheat production systems.

### 4.2 Effects of nitrogen application on senescence characteristics of flag leaves and plant DMA and translocation

High nitrogen supply enhances the activity of cellular enzymatic defense systems, as confirmed by our findings. Leaf malondialdehyde (MDA) content was lower under N210 and N270 treatments, while superoxide dismutase (SOD) activity and soluble protein concentration remained high in both growing seasons (**Figure 3**). These observations indicate a significantly improved redox defense status in plants, effectively mitigating reactive oxygen species (ROS)-induced damage. This aligns with reports that optimized nitrogen fertilization promotes antioxidant compound accumulation and strengthens plant protection against peroxidation (Wang et al., 2022, 2024). The parallel trends in photosynthetic parameters and SPAD values observed in flag leaves reinforce these conclusions (**Figure 2**), demonstrating that nitrogen-enhanced antioxidant capacity protects photosynthetic machinery from oxidative degradation. Nitrogen-deficient plants paradoxically enhance peroxidative protection through compensatory antioxidant compound accumulation—a stress response mechanism activated under nutrient limitation. Conversely, excessive nitrate or ammonium nitrogen induces nutritional stress, potentially explaining the plateau in leaf antioxidant capacity under N270 (270 kg ha^−1^) treatment. This biphasic response reflects threshold effects in nitrogen signaling.

Our results demonstrate that nitrogen-mediated regulation of dry matter accumulation (DMA) significantly influences grain dry matter accumulation (**Figure 6**). N270 promoted DMA during overwintering and jointing stages, while N210 and N270 showed no significant differences at flowering and maturity. This pattern likely arises from N270’s stimulation of tillering capacity through increased cytokinin synthesis in shoot apical meristems during early growth phases, followed by substantial mortality of late-formed inferior tillers post-jointing due to carbohydrate competition (Hao et al., 2023). Enhanced photoassimilate conversion to grains constitutes a critical yield-enhancement strategy. In our study, N210 increased post-anthesis assimilate transport while optimizing dry matter partitioning to grains at maturity. In contrast, N270 allocated a greater proportion of dry matter to vegetative organs, constraining potential grain yield increases. This aligns with the report by Si et al. (2020), who observed yield declines when nitrogen applications exceeded 240 kg ha^−1^. Under low nitrogen inputs, insufficient nitrogen fails to meet reproductive growth demands, potentially inducing premature leaf senescence during grain filling through ABA-mediated senescence signals. Conversely, excessive nitrogen delays senescence by maintaining cytokinin levels and downregulating senescence-associated genes, promoting nonstructural carbohydrate accumulation in straw at the expense of grain yield (Du et al., 2024; Meng et al., 2024). This nitrogen-dependent source-sink reprogramming highlights the importance of balanced nitrogen management in synchronizing assimilate production, transport, and utilization processes.

### 4.3 Effects of nitrogen application on grain filling and grain yield

Increased nitrogen (N) application significantly enhances vascular bundle architecture, improves assimilate transport efficiency, and optimizes photosynthetic allocation to grains, ultimately boosting the grain-filling rate and yield (Ren et al., 2021). In this two-year field experiment, we observed significant positive correlations between thousand-grain weight (TGW), Tmax (days required to reach maximum grain-filling rate), and Wmax (grain weight at maximum filling rate) (**Figure 8**). The parameters Tmax, T (filling duration), and D (active filling period) increased with elevated N input, whereas Wmax and Vmax (mean filling rate) initially increased before showing a slight decline. These findings suggest that a high N supply prolongs the grain-filling duration while reducing the filling rate, likely due to enhanced nitrogen metabolism and carbohydrate consumption in plant tissues, coupled with reduced carbohydrate translocation to grains (Liu et al., 2023; Ravier et al., 2017). Consistent with previous studies, the spike number exhibited an incremental response to the N application rate. Insufficient N availability accelerates plant senescence, shortens the grain-filling period, and reduces grain weight (GW) and yield. Conversely, excessive N input decreases filling intensity, extends filling duration, and paradoxically compromises GW in winter wheat (Liu et al., 2021; Yue et al., 2022). Grains per spike and TGW initially increased but subsequently declined with increasing N applications. Across both growing seasons, the maximum values for grains per spike, TGW, and yield were achieved at the N210 level. A further increase in N270 resulted in significant reductions in these parameters (**Table 3**). In summary, maintaining appropriate soil N effectiveness and avoiding over-fertilization under water-saving irrigation are key to improving crop nutrition and achieving high wheat yields. These findings emphasize the need to balance N inputs with crop requirements through precise management strategies that synchronize N release with key developmental transitions.

### 4.4 Limitations and perspectives

Our findings have demonstrated that the most effective nitrogen (N) application rate for achieving wheat yield enhancement is 210 kg ha^−1^. While this study primarily focused on the wheat growing season, it remains crucial to acknowledge the residual nitrogen effects from the maize season when optimizing nitrogen management in wheat–maize double-cropping systems. Constrained by field experiment limitations, our investigation included only four Newton application treatments. This restricted range may be insufficient to capture the potential nonlinear relationships between fertilizer application and yield responses. The relatively large intervals between the experimental treatment settings may have overlooked the subtle yield effects associated with critical nitrogen application thresholds.

The conclusions presented in this study are derived from specific soil types and climatic conditions of the NCP. Therefore, the generalizability of these findings to regions with distinct environmental conditions requires careful consideration through integration with long-term experimental data from diverse geographical contexts. Although in this study, we prioritized wheat yield improvement, it did not fully evaluate the environmental externalities associated with increased nitrogen inputs, including water eutrophication, soil contamination, and elevated greenhouse gas emissions. Implementing systematic environmental impact assessments of nitrogen fertilization practices could facilitate the development of more ecologically sustainable agricultural policies and provide valuable insights into optimizing nitrogen application strategies to achieve regionally adapted sustainable crop production systems.

## 5 Conclusions

This two-year study showed that nitrogen (N) application significantly regulates post-anthesis physiological processes and dry matter partitioning in winter wheat. Elevated N rates (N210 and N270) enhanced flag leaf functionality compared with the low N treatment (N150), as shown by increased SPAD values (16.76–19.64%), photosynthetic capacity (19.59–20.57%), and antioxidant enzyme activity (8.08–10.35%), thereby prolonging leaf functional duration. Both N210 and N270 significantly increased sucrose content and SPS activity in flag leaves post-anthesis, concomitant with enhanced dry matter accumulation and translocation during the grain-filling phase. Over the two experimental years, N210 had the highest grain yield (9414.55–9616.98 kg ha^−1^) and harvest index (47.65–48.89%). This is attributable to its ability to accelerate grain-filling rates, extend the active grain-filling period, and optimize assimilate allocation to grains at maturity. An application rate of 210 kg N ha^−1^ demonstrated optimal yield potential under supplemental irrigation. Meanwhile, excessive N (N270) failed to further improve productivity despite the higher physiological activity. These findings provide actionable strategies for nitrogen management in winter wheat cultivation in semi-arid regions with similar climatic conditions.

## Data Availability

The original contributions presented in the study are included in the article/**Supplementary Material**. Further inquiries can be directed to the corresponding author.
