# Supplementary material for: Optimizing nitrogen application strategies can improve grain yield by increasing dry matter translocation, promoting grain filling, and improving harvest indices
Source: Front Plant Sci. 2025 Apr 30;16:1565446. doi: 10.3389/fpls.2025.1565446 (PMC12075212; doi:10.3389/fpls.2025.1565446)
Supplement: Supplementary file 1 [file Table1.docx]

Supplementary Material

**Table S1** The irrigation and nitrogen fertilizer regimes in this study

| Treatment | | Irrigation (mm) | | |  | Nitrogen fertilizer (kg·ha^-1^) | | |
| --- | --- | --- | --- | --- | --- | --- | --- | --- |
|  |  | Jointing | Anthesis | Total |  | Pre-sowing | Jointing | Total |
| 2020-2021 | N0 | 45.26 | 48.96 | 94.22 |  | — | — | — |
|  | N150 | 50.52 | 54.46 | 104.98 |  | 75 | 75 | 150 |
|  | N210 | 57.46 | 60.48 | 117.94 |  | 105 | 105 | 210 |
|  | N270 | 63.79 | 65.42 | 129.21 |  | 135 | 135 | 270 |
| 2021-2022 | N0 | 48.50 | 52.19 | 100.69 |  | — | — | — |
|  | N150 | 53.74 | 57.67 | 111.42 |  | 75 | 75 | 150 |
|  | N210 | 60.67 | 63.68 | 124.35 |  | 105 | 105 | 210 |
|  | N270 | 66.98 | 68.61 | 135.59 |  | 135 | 135 | 270 |

Note: N0, N150, N210 and N270, the nitrogen application rates are 0, 150, 210 and 270 kg·ha^-1^, respectively.
